# The Absence of Caspase-8 in the Dopaminergic System Leads to Mild Autism-like Behavior

**DOI:** 10.3389/fcell.2022.839715

**Published:** 2022-04-05

**Authors:** I. Suárez-Pereira, I. García-Domínguez, L. Bravo, M. Santiago, J. García-Revilla, A. M. Espinosa-Oliva, I. M. Alonso-Bellido, C. López-Martín, E. M. Pérez-Villegas, J. A. Armengol, E. Berrocoso, J. L. Venero, R. M. de Pablos, R. Ruiz

**Affiliations:** ^1^ Centro de Investigación Biomédica en Red de Salud Mental (CIBERSAM), Instituto de Salud Carlos III, Sevilla, Spain; ^2^ Neuropsychopharmacology and Psychobiology Research Group, Instituto de Investigación e Innovación en Ciencias Biomédicas de Cádiz, INiBICA, University of Cádiz, Cádiz, Spain; ^3^ Departamento de Bioquímica y Biología Molecular, Facultad de Farmacia, Instituto de Biomedicina de Sevilla-Hospital Universitario Virgen del Rocío/CSIC/Universidad de Sevilla, Sevilla, Spain; ^4^ Departamento de Fisiología, Anatomía y Biología Celular, Universidad Pablo de Olavide, Sevilla, Spain

**Keywords:** autism spectrum disorder, dopaminergic system, caspase 8, electron microscopy, synapsis, behavioral test

## Abstract

In the last decade, new non-apoptotic roles have been ascribed to apoptotic caspases. This family of proteins plays an important role in the sculpting of the brain in the early stages of development by eliminating excessive and nonfunctional synapses and extra cells. Consequently, impairments in this process can underlie many neurological and mental illnesses. This view is particularly relevant to dopamine because it plays a pleiotropic role in motor control, motivation, and reward processing. In this study, we analyze the effects of the elimination of caspase-8 (CASP8) on the development of catecholaminergic neurons using neurochemical, ultrastructural, and behavioral tests. To do this, we selectively delete the CASP8 gene in cells that express tyrosine hydroxylase with the help of recombination through the Cre-loxP system. Our results show that the number of dopaminergic neurons increases in the substantia nigra. In the striatum, the basal extracellular level of dopamine and potassium-evoked dopamine release decreased significantly in mice lacking CASP8, clearly showing the low dopamine functioning in tissues innervated by this neurotransmitter. This view is supported by electron microscopy analysis of striatal synapses. Interestingly, behavioral analysis demonstrates that mice lacking CASP8 show changes reminiscent of autism spectrum disorders (ASD). Our research reactivates the possible role of dopamine transmission in the pathogenesis of ASD and provides a mild model of autism.

## 1 Introduction

Autism spectrum disorder (ASD) is a heterogeneous neurodevelopmental disorder with a prevalence in US of 18.5 per 1,000 people in the general population (Maenner et al., 2020). The main symptoms of ASD include disturbances in social interactions and communication and repetitive stereotyped behavior patterns. In addition to these cardinal symptoms, patients with autism may suffer from other physiological and psychiatric comorbidities, including anxiety, intellectual disabilities, motor disorders, gastrointestinal problems, attention and language impairment, and hyper or hyporreactivity (Lai et al., 2014). All this, along with the scarcity of effective treatments, makes ASD a disorder that generates a significant socioeconomic burden.

The lack of effective treatments for ASD reflects the limited understanding of the biology underlying this neurodevelopmental disorder. Increasing evidence suggests that behind the autism pathophysiology there are neurochemical and neuroanatomical events that occur relatively early in the development of the central nervous system (CNS). In this sense, neurotransmitters and neuropeptides play a fundamental role in normal brain development and influence synaptogenesis and neuronal cell migration, differentiation, apoptosis, and synaptic pruning that contribute to the regulation of memory, behavior, and motor activity (Quaak et al., 2013). Therefore, dysfunction of the neurotransmitter system can cause impairments in brain development processes, determining autism. Several neurotransmitters have been involved in ASD, including GABA, glutamate, serotonin, and acetylcholine, among others. Many studies also suggest that ASD could be associated with dopaminergic dysfunction and postulate that dopamine (DA) imbalances in certain areas of the brain may lead to autistic behavior (Pavǎ;l and Miclutia, 2021; Kosillo and Bateup, 2021; Dichter et al., 2012). Accordingly, social deficits in patients with ASD could be determined by dysfunction of the mesocorticolimbic circuit, while dysfunction of the nigrostriatal circuit could lead to stereotyped behaviors (Pavǎ;l, 2017). The involvement of the dopaminergic system in ASD has been reinforced by the description of some polymorphisms of genes related to the dopaminergic system associated with ASD. Examples include the plasma membrane monoamine transporter (SLC29A4), several DA receptors (DRD1, DRD2, DRD3, and DRD4), the DA transporter (DAT), and genes encoding proteins important for DA synthesis and catabolism (for a review, see (Kosillo and Bateup, 2021)). Furthermore, the most common single gene mutation associated with ASD is fragile X mental retardation 1 gene (FMR1), which codes a key messenger for modulation of DA (Wang et al., 2008).

We have recently described a new model of experimental autism in animals lacking the *CASP3* gene specifically in the dopaminergic system (García-Domínguez et al., 2021). These animals show dopaminergic alterations and impaired social interaction, restrictive interests, and repetitive stereotypies, which are considered the core symptoms of ASD. These results highlight the role of caspases in the development of the dopaminergic system during embryogenesis. Caspase-3 (CASP3) can be activated by two signaling mechanisms: 1) the cell-intrinsic pathway, initiated by some cell stress events and mediated by proteins of the Bcl-2 family, and 2) the cell-extrinsic pathway, triggered by extracellular signals that specifically bind to death receptors on the surface of the target cell (Cory and Adams, 2002) (Nair et al., 2014). Here, we explore the role of caspase-8 (CASP8), the best known initiator caspase involved in the extrinsic pathway, in the development of the dopaminergic system and its possible implication in ASD. Using several molecular, histological, and behavioral approaches, we found that CASP8 participates in the development of the dopaminergic system and that its absence led to mild ASD-like behavior.

## 2 Material and Methods

### 2.1 Animal Model

*Caspase-8* ^f/f^ C57BL/6 mice with the *CASP8* allele floxed at exon 3 were generously provided by Prof. Steven M. Hedrick (University of California, San Diego). C57BL/6 mice containing an IRES-Cre recombinase under the control of the tyrosine hydroxylase (TH) promoter were kindly provided by José López Barneo (Instituto de Biomedicina de Sevilla). Both colonies were maintained at the Centre of Production and Animal Experimentation of the University of Seville. The animals were housed at constant room temperature (RT) of 22 ± 1°C and relative humidity (60%), with a 12-h light-dark cycle and *ad libitum* access to food and water. Experiments were carried out in accordance with the Guidelines of the European Union Directive (2010/63/EU) and Spanish regulations (BOE 34/11370-421, 2013) for the use of laboratory animals; the study was approved by the Scientific Committee of the University of Seville.

**FIGURE 1:**
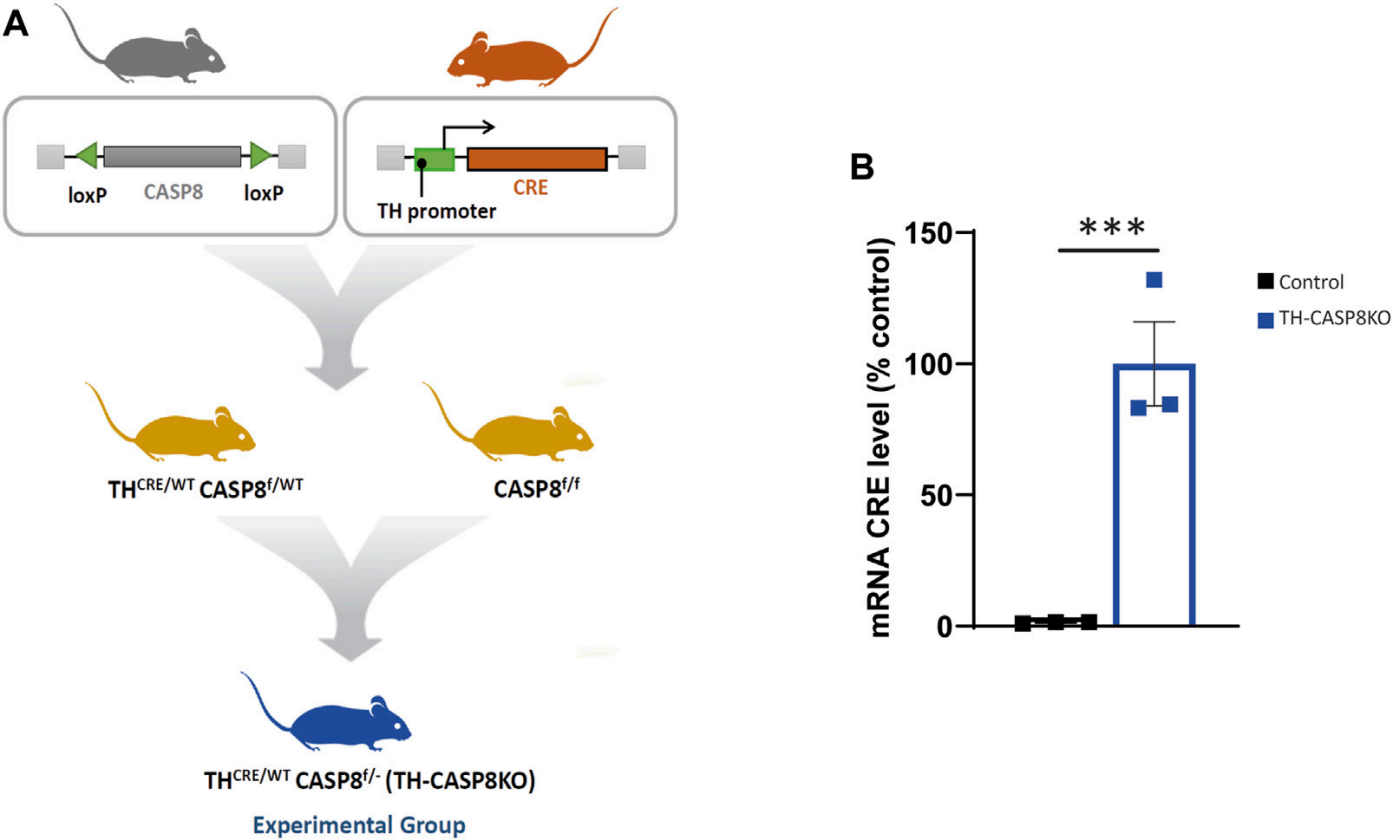
Generation of conditional TH-CASP8KO mice. **(A)** Crossing strategy for the generation of TH-CASP8KO mice (experimental group). **(B)** qPCR analysis of the mRNA expression of CRE. Data are expressed as mean ± SEM of n = 3 mice/group. The two-tailed unpaired *t* test was used. ***, *p* < 0.01 *versus* control mice.

Under our experimental conditions, the *Cre* gene is preceded by the encephalomyocarditis virus IRES by a knock-in strategy (Lindeberg et al., 2004)). This enables the expression of bicistronic mRNA encoding both TH and Cre (Lindeberg et al., 2004). This strategy has provided very efficient genomic recombination in TH-expressing cells. However, germ cells from both sexes were also shown to exhibit recombination. This feature was used as a novel strategy to knock out *CASP8* in one allele by crossing TH^Cre/wt^CASP8^wt/f^ with CASP8^f/f^ to generate TH^Cre/wt^CASP8^f/-^ mice (experimental mouse, hereafter TH-CASP8KO). This strategy ensures high deletion of the *CASP8* gene in cells expressing TH (Figure 1A). Three to six-month-old male TH-CASP8KO and age-matched male C57BL/6 mice (control) (20–25 g) were used in our experiments.

### 2.2 Behavioral Testing

Behavioral experiments were conducted in dedicated behavioral testing rooms during the standard light phase, usually between 09:00 and 15:00 h. Mice were brought to a holding room in the hallway of the testing area at least 30 min prior to behavioral testing. All task equipment was thoroughly cleaned with 70% ethanol between trials to remove any olfactory signals. Complete blind rating on tasks that were scored in real time, as well as when scoring was performed from videotaped sessions. At least six mice per genotype were tested in all behavioral assays. Additional mice underwent behavioral testing on more than one, but not all, assays. Each animal performed different behavioral tests, but not all of the proposals, always allowing them a rest between tests and establishing an ascending stress testing order. Therefore, the following testing order was used in two independent experimental rounds: 1/locomotor activity/open field, rotarod, tail suspension test and prepulse inhibition of the acoustic startle response; 2/nesting, olfactory test, hot plate, and social behavior. Experimental mice derived from two different litters.

#### 2.2.1 Locomotor Activity

Spontaneous activity was measured during 30 min (Ramos-Rodriguez et al., 2013). Mice were placed in a square arena (45 × 45 cm) enclosed by continuous, 35-cm-high, opaque walls, located in a room with constant dim lighting (estimated about 10–20 lux) and with constant background noise (i.e. the behavioral tests were performed in silence, avoiding making noise except for the unavoidable room background noise, such as the ventilation or light system). All sessions were recorded and evaluated using the SMART 3.0 video tracking system (Panlab). Activity in arbitrary units (mobility respect to reference, video snapshot, by the software) was collected in 5 min intervals and total time. In addition, resting time and the number of grooming and rearing events were recorded as a measure of motor stereotypies.

The open field (OF) test was carried out in a 2,025 cm^2^ enclosure for 10 min, the center of which was defined as a square that covered 50% of the total diameter of the OF arena. The time spent in the central area was monitored over a 10 min test period using the SMART 3.0 video tracking system (Panlab).

#### 2.2.2 Rotarod

Motor coordination and balance were evaluated in a rotarod apparatus (Panlab). Mice were placed on the rotarod and two tests were performed: at constant speed (5 rpm) for a maximum of 5 min (3 trials with 15 min rest between trials), and at accelerated speed (4–40 rpm in 5 min, only one trial) (Nóbrega et al., 2013). The researcher counted the number of falls and the latency of the fall was automatically recorded by the apparatus, respectively.

#### 2.2.3 Tail Suspension Test

Mice were individually and securely suspended for 6 min at the distal end of the tail from an aluminum hook raised 20 cm above the floor using adhesive tape. The test sessions were recorded and analyzed blindly by a researcher (Berrocoso et al., 2013). The analysis procedure used was a time sampling technique, whereby the predominant behavior was scored in each 5-s period of the 360-s test. The behaviors rated were: 1) immobility–a mouse was judged to be immobile when it hung by its tail without engaging in any active behavior; 2) swinging–a mouse was judged to be swinging when it continuously moved its paws in the vertical position while keeping its body straight and/or it moved its body from side to side; 3) curling–a mouse was judged to be curling when it engaged in active twisting movements of the entire body; 4) clasping–a mouse was judged to be clasping when it showed hindlimb grasping behavior towards the abdomen.

#### 2.2.4 Prepulse inhibition of the acoustic startle response

Mice were tested for ASR and PPI using the startle reflex system (Panlab) (Urigüen et al., 2013). Background noise (65 dB) was present throughout the session. The startling stimulus was a broadband acoustic pulse with an intensity of 120 dB and a duration of 30 ms and was administered alone (“pulse-alone” trials) or paired with the prior presentation of a 30 ms duration prepulse (“prepulse” trials). The intensity of the prepulse stimulus was set to 3, 6, or 12 dB above background noise and delivered with an interstimulus interval of 120 ms (onset-to-onset). A testing session contained a habituation period, a baseline with 5 pulse-alone, a total of 40 test trials (10 pulse-alone and 10 prepulse trials at each prepulse intensity) and a final repetition of baseline. The inter-trial intervals ranged from 10–20 s. The magnitude of the startle was calculated as the average of the startle responses to the respective pulse trials. PPI was calculated according to the formula: %PPI = (1—(startle response for prepulse + pulse trials/startle response for pulse alone trials)) × 100.

#### 2.2.5 Olfatory Test

Mice were accustomed to the flavor of a new food (Kellogg’s Chocolate Cereal bar) for 3 days prior, in order to avoid food neophobia on the day of the test. Olfactory ability was evaluated after a period of food deprivation. All food was removed from the home cage 16–20 h before the test. On the day of the test, each mouse was placed in a clean cage containing 3 cm of bedding and allowed to explore for 5 min. The animal was removed from the cage and 1 cm^3^ of cereal bar was buried in the cage bedding, approximately 1 cm below the surface of the litter (Moy et al., 2008). The latency to find the food was measured.

#### 2.2.6 Hot Plate

Response to an acute thermal stimulus was measured using the hot plate test (Berrocoso and Mico, 2009). The mouse was placed on a flat, black metal surface (Ugo Basile, Socrel DS-37) maintained at 55°C and surrounded by a square transparent plexiglass barrier to prevent jumping off. The latency to the first paw lick, jump, or vocalization was measured by an observer using a foot pedal-controlled timer. A maximum cut-off time of 30 s was used to prevent the risk of tissue damage to the paws.

#### 2.2.7 Social behavior

This test was carried out in an experimental cage (40 × 24 × 18 cm) with bedding. For habituation, subject mice were first placed in the middle of the cage and allowed to explore for 10 min. For the novelty preference test, an empty wire cage (wire cups; diameter, 7.7 cm; height, 10 cm) was placed on the side of the chamber, and then the subject mice were allowed to explore for 10 min. For the sociability test, a new mouse (stranger, different strain, age matched male C3H/HeJ mice) was enclosed on the wire cage and placed in the same side of the chamber, and again the subject mice were allowed to explore for 10 min. The time spent sniffing each wire cage (empty and stranger house) and in each of the side chambers and the latency to the first contact was measured. A mouse was considered to be sniffing the wire cage when its head was facing the cage within 1 cm (Moretti et al., 2005).

#### 2.2.8 Nesting

Nest building and utilization were assessed during 2 days of single housing (Moretti et al., 2005). The nesting material, two cotton wafers (5 cm^3^), was introduced into the cage. After 1 and 24 h, the quality was recorded together with the height of the nest. The quality of the nest was measured using the following scale: 0) nesting material unmodified; 1) flat nest with partially shredded nest material; 2) shallow nest with shredded material but lacking fully formed walls; 3) nest with well-developed walls; and 4) nest in the shape of a cocoon with partial or complete roof.

### 2.3 Immunohistochemistry

At the end of the behavioral experiments, animals were perfused through the heart under deep anesthesia (isoflurane) with 150–200 ml of 4% paraformaldehyde in phosphate buffer, pH 7.4. The brains were removed and then cryoprotected serially in sucrose dissolved in phosphate-buffered saline (PBS), pH 7.4, first (24 h) in 10% sucrose, next (24 h) in 20% sucrose, and finally in 30% sucrose until they sank (2–5 days). Brains were then frozen in isopentane at −20°C, and sections of 30 μm thickness were cut on a cryostat at −20°C and conserved at −20°C. TH expression was evaluated in the dopaminergic substantia nigra pars compacta (SNpc), the ventral tegmental area (VTA), and the striatum. After washing with PBS (5-min, 3 times), free floating sections were subjected to an inactivation of endogenous peroxidase with PBS containing 0.3% hydrogen peroxide (30 min). Sections were washed 3 times (5 min each) in PBS containing 2.5% Triton X-100 (PBS-T 2.5%) and incubated for 2 h with PBS-T 2.5% containing 2.5% BSA. Then, sections were incubated with rabbit anti-TH at 4°C (1:500, Invitrogen, OPA1-04050, for 48 h for optical analysis; 1:2000, Sigma, T8780, for 24 h, for stereological analysis) dissolved in PBS-T 2.5% containing 2.5% BSA. Subsequently, sections were incubated for 1.5 h with a biotinylated donkey anti-rabbit (1:500, Jackson Immunoresearch Europe, 711-065-152, for optical analysis) or goat anti-rabbit (1:200, Vector, BA-1000, for stereological analysis) dissolved in PBS-T 2.5% containing 1% BSA and its additions was preceded by 5-min rinses in PBS-T 2.5%. Then, sections were washed again in PBS and incubated for another hour in an avidin-biotin complex (ABC, Vector PK-6100) conjugated with horseradish peroxidase (1:500). After washing again 3 times in PBS, visualization of immunostaining was achieved using the 3,3′-diaminobenzidine tetrahydrochloride (DAB) reaction (1 min in 0.2 M Tris buffer containing 0.1% DAB and 0.001% hydrogen peroxide). Sections were mounted on gelatine-coated slides, cleared in xylene, and coverslipped with DPX (Sigma-Aldrich, 06522).

For optical density analysis, for each structure, 1 of every 5 sections of 40 µm was evaluated. The number of TH-immunoreactivity cells (TH-IR) was captured on an Olympus BX60 microscope. An experimenter blind to conditions manually counted the labelled cell bodies (TH-IR) in an average of 3–5 sections per animal (n = 6–7). The optical density of the expression of TH was calculated after manually delimiting the region of interest and applying the mean intensity less the background noise of each section (3–5 sections) using Fiji ImageJ (W. Rasband, National Institutes of Health) and therefore expressing in arbitrary units (a.u.).

For stereological analysis, sections of 30 µm thickness were evaluated. Analysis was made in a bounded region of the SNpc with a length of 300 microns in the anterior–posterior axis between 5.35 and 5.65 mm with respect to bregma. In each case, 5 sections per animal were used, with random starting points and systematically distributed through the anterior–posterior axis of the analyzed region. The number of TH^+^Nissl^+^ and TH^−^Nissl^+^ neurons in the SNpc was estimated using a fractionator sampling design (Gundersen et al., 1988). Counts were made at regular predetermined intervals (x = 150 µm and y = 200 µm) within each section. An unbiased count frame of the known area (40 μm × 25 µm = 1,000 μm^2^) was superimposed on the image of the tissue section under a 100x oil immersion objective. Therefore, the area sampling fraction is 1,000/(150 × 200) = 0.033. The entire z-dimension of each section was sampled; therefore, the section thickness sampling fraction was 1. In all animals, 30-µm sections, each 100 µm apart, were analyzed; thus, the fraction of sections sampled was 30/100 = 0.30. The total number of TH^+^Nissl^+^ and TH^−^Nissl^+^ neurons in the analyzed region was estimated by multiplying the number of neurons counted within the sample regions by the reciprocals of the area sampling fraction and the fraction of section sampled.

### 2.4 Real-Time RT-PCR

The animals used for RT-PCR were sacrificed by decapitation. SN was dissected from each mouse, snap frozen in liquid nitrogen, and stored at −80°C. Total RNA was extracted from mouse SN of different groups using the RNeasy^®^ kit (Qiagen). cDNA was synthesized from 1 μg of total RNA using the Revert Aid First Strand cDNA Synthesis Kit (Thermo Fisher Scientific) in 20 μL reaction volume as described by the manufacturer.

Real-time PCR was performed using 5 μL SensiFAST™ SYBR NO-ROX KIT (Bioline, United States), 0.4 μL of each primer and 4.2 μL cDNA to obtain a final reaction volume of 10 μL for a 384-well plate. Controls were carried out without cDNA. Amplification was run in a Lightcycler^®^ 480 Instrument II (Roche) thermal cycler at 95°C for 2 min followed by 40 cycles consisting of a denaturation phase for 5 s at 95°C, followed by a second phase of hybridization at 65°C for 10 s, and a final phase of elongation at 72°C for 20 s. The process was ended with a final step of 7 min at 72°C. Analysis confirmed a single PCR product. β-actin served as a reference gene and was used for sample normalization. The cycle in which each sample crossed a fluorescence threshold (Ct value) was determined and the triplicate values for each cDNA were averaged. The primer sequences are: TH (F:5′- GGC​TAT​GCT​CTC​CCT​CAC​G and R:5′- CTT​CTC​TTT​GAT​GTC​ACG​CAC​G, CRE (F:5′-GTCGAGCGATGGATTTCCG and R:5′-GTTGATAGCTGGCTGGTGG) and β-actin (F:5′- CTG​AAG​GGC​CTC​TAT​GCT​AC and R: 5′- CCA​CAG​TAC​CGT​TCC​AGA​AG).

### 2.5 Measurement of DA, DOPAC, and HVA

Strital DA and its metabolites (3,4-dihydroxyphenylacetic acid (DOPAC) and homovanillic acid (HVA)) were analyzed by HPLC equipped with a vwr-Hitachi Elite Lachrom L-2130 pump in conjunction with a glassy carbon electrode set at −550 mV (DECADE II, ANTEC). A Merck Lichrocart cartridge (125 mm × 4 mm) column filled with Lichrospher reverse phase C_18_ 5 µm material was used. The mobile phase consisted of a mixture of 0.05 M sodium acetate, 0.4 mM 1-octanesulfonic acid, 0.3 mM Na_2_EDTA and 70 ml methanol/l, adjusted to pH 4.1 with acetic acid. All reagents and water were HPLC grade. The flow rate was 1.0 ml/min. The measurement of all the molecules in the fresh tissue was performed according to the method previously described (Ismaiel et al., 2016). The concentration of striatal DA, DOPAC, and HVA was calculated with the aid of an eDAQPowerChrom 280 software.

### 2.6 Microdialysis

Microdialysis in the corpus striatum was performed under deep anesthesia with isoflurane with an I-shaped cannula (Santiago and Westerink, 1990). The exposed tip of the dialysis membrane was 2 mm. The dialysis tube (ID: 0.22 mm; OD: 0.31 mm) was prepared from a polyacrylonitrile/sodium methalyl sulfonate copolymer (AN 69, Hospal, Barcelona, Spain). The stereotaxic coordinates used were AP = +0.4 mm; L = ±2 mm, DV = −4.0 mm to conform to a mouse brain atlas (Paxinos and Franklin, 2001); Figure 3D.

Perfusion experiments were carried out at 24 and 48 h after probe implantation. Microdialysis and subsequent chemical analysis were performed using an automated online sample injection system (Westerink et al., 1987). The corpus striatum was perfused at a flow rate of 3.0 μl/min, using a microperfusion pump (model 22, Harvard Apparatus, South Natick, MA, United States), with a Ringer solution containing (in mM): NaCl, 140; KCl, 4.0; CaCl_2_, 1.2; and MgCl_2_, 1.0. With the help of an electronic timer, the injection valve was held in the load position for 15 min, during which the sample loop (40 μl) was filled with dialysate. The valve was then automatically switched to the injection position for 15 s. This procedure was repeated every 15 min, the time needed to record a complete chromatogram.

### 2.7 Electron Microscopy

Two control and two TH-CASP8KO mice were deeply anesthetized with pentobarbital (80 mg/kg i. p.) and perfused intracardially with ice-cold 1% paraformaldehyde, 1% glutaraldehyde and 0.02% Ca Cl_2_ in 0.12 M phosphate buffer (PB, pH 7.2-7.4) fixative. The brains were left within the cranium and stored overnight in the fixative solution at 4°C. After dissection, the brains were cut into 500-1,000 μm thick coronal slabs, post-fixed in 2% OsO_4_ in, stained in block with 1% uranyl acetate in 70% ethanol, dehydrated and flat embedded in Spurr’s epoxy resin. Semi-thin sections (1.5 μm) and ultrathin (60–70 nm) sections were obtained on a Leica UC6 ultramicrotome. Selected regions of semi-thin sections containing the dorsal striatum were trimmed and cut. Ultrathin section ribbons were collected, one ribbon per grid, on copper grids (150 and 300 mesh) and viewed without counterstaining in a Zeiss Libra EM at 80 kV (CITIUS, University of Seville).

Quantitative analysis of axospinous synapses was previously described (García-Domínguez et al., 2021). Briefly, mosaics of the dorsal striatum of 3 × 3 (85 μm^2^ area) microphotographs were obtained with the multiple image acquisition application of the Olympus iTEM software^®^. Mosaics were stitched with the Image Composite Editor software (Microsoft). To ensure that the counting of axospinous synapses was unbiased and that the sections used for it were different, the cutting distance between the section ribbons was 3 μm. All counts were performed with Fiji ImageJ software (W. Rasband, National Institutes of Health).

### 2.8 Data Analyses and Statistics

Data were analyzed with GraphPad PrismTM 4.0 (GraphPad Software, San Diego, CA, United States). Results are expressed as mean ± SEM values. Data were analyzed by using unpaired Student’s t-test or one-way analysis of variance tests followed by Bonferroni. The level of significance was set to *p* < 0.05.

## 3 Results

### 3.1 TH^Cre^CASP8^f/-^ Conditional Mice

To investigate the role of CASP8 in the development of dopaminergic neurons, a conditional mouse with dopaminergic specific depletion of CASP8 was generated (see the Materials and Methods section and Figure 1A). First, we checked that the mice effectively presented a real expression of CRE in midbrain dopaminergic neurons by using the qPCR technique. Increase in CRE mRNA expression was clear in dopaminergic neurons belonging to SNpc in the experimental model compared to control CRE negative mice (Figure 1B; *p* < 0.001) confirming that the lox-CRE system to generate conditional deletion of CASP8 in TH^+^ neurons works in this model.

### 3.2 *CASP8* Removal in TH Neurons Alters the Dopaminergic System in TH-CASP8KO Mice

**FIGURE 2:**
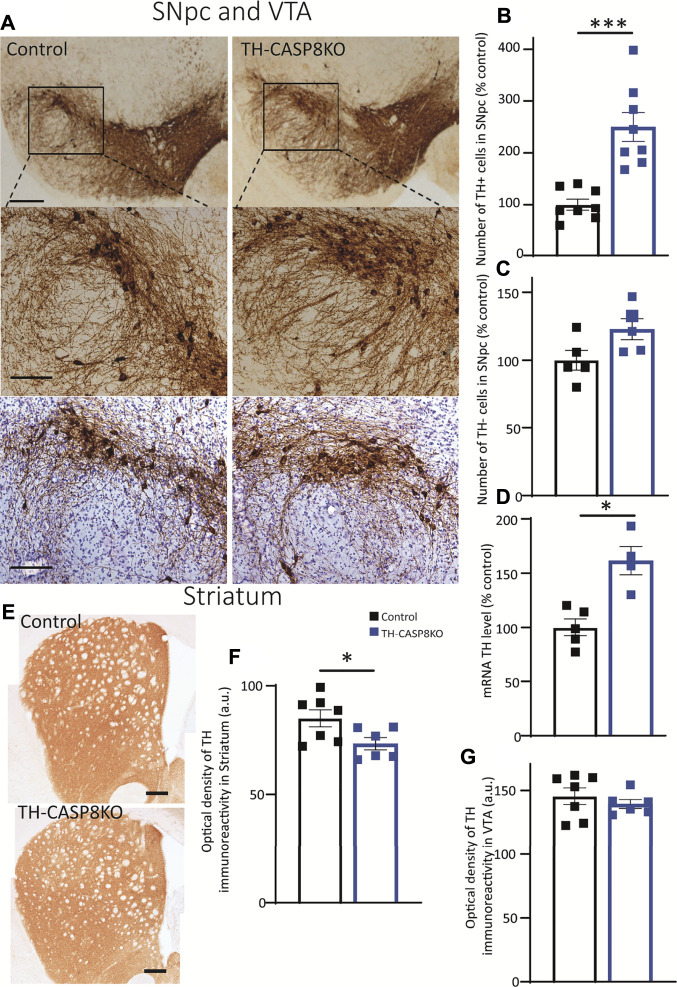
Effect of *CASP8* deletion on TH expression. **(A)** Coronal sections of SN from control and TH-CASP8KO animals showing immunoreactivity against TH. A more intense staining can be observed in the TH-CASP8KO animal. Lower panels show the immunohistoquemistry costained with Nissl. The deletion of *CASP8* generated differences in the percentage of TH^+^ cells (n = 8 mice/group) **(B)** and in the expression level of TH mRNA (n = 4 mice/group) **(D)** in SNpc. **(C)** Quantification of TH-negative-Nissl-positive cells in the SN of control and TH-CASP8KO animals. **(G)** Optical density of the immunoreactivity in VTA, expressed in a. u. **(E)** Coronal sections of striatum of control and TH-CASP8KO animals showing immunoreactivity against TH. The deletion of *CASP8* decreased the optical density of TH immunoreactivity in the striatum. Scale bar 100 and 200 µm. **(F)**. Data are expressed as mean ± SEM of n = 7-6 mice/group. The two-tailed unpaired *t* test was used. *, *p* < 0.05; ***, *p* < 0.05 *versus* control mice.

During postnatal development, the existence of naturally occurring death within SN dopaminergic neurons has been reported in both rats (Janec & Burke, 1993) and mice (Jackson-Lewis et al., 2000). However, evidence supporting the key role of CASP8, as an apoptotic protein that drives the development death of dopaminergic neurons, is lacking. Therefore, our experimental mice emerge as an excellent tool to analyze the involvement of CASP8 in the maturation of the dopaminergic system. Consequently, we first used the immunohistochemistry approach to measure the number of TH^+^ neurons in the SNpc in TH-CASP8KO in comparison with control group (Figures 2A,B). This analysis reported an increase in the number of TH^+^ neurons in the SNpc of TH-CASP8KO mice *versus* the control group (Figure 2A). Stereological analysis of TH neurons reported a significant increase of three times the number of TH^+^ neurons in the entire SNpc in TH-CASP8KO animals (Figure 2B; *p* < 0.001). We also wanted to know if the absence of CASP8 could affect non dopaminergic neurons in the SNpc. To test this, we performed a TH immunostaining along with a cresyl violet staining. Our results showed no statistical difference in the number of non-TH labeled neurons in the SNpc (Figure 2A lower panels and 2C), which ruled out a possible phenotypic conversion of non-TH to TH labelled neurons. In the same way, the level of TH mRNA measured by qPCR showed an increase of ∼49% in TH-CASP8KO *versus* control animals (Figure 2D; *p* < 0.05). However, no differences were found in VTA (Figure 2G). The striatum is the brain region where the fibers of the SNpc dopamine neurons project. Therefore, our next step was to study whether the increase in TH^+^ neurons was accompanied by an increase in projected fibers. Unexpectedly, a ∼14% decrease in TH immunoreactivity was found in TH-CASP8KO mice (Figures 2E,F; *p* < 0.05).

### 3.3 *In vivo* Levels of DA and Its Metabolites in the Striatum Are Altered in TH-CASP8KO Mice

**FIGURE 3:**
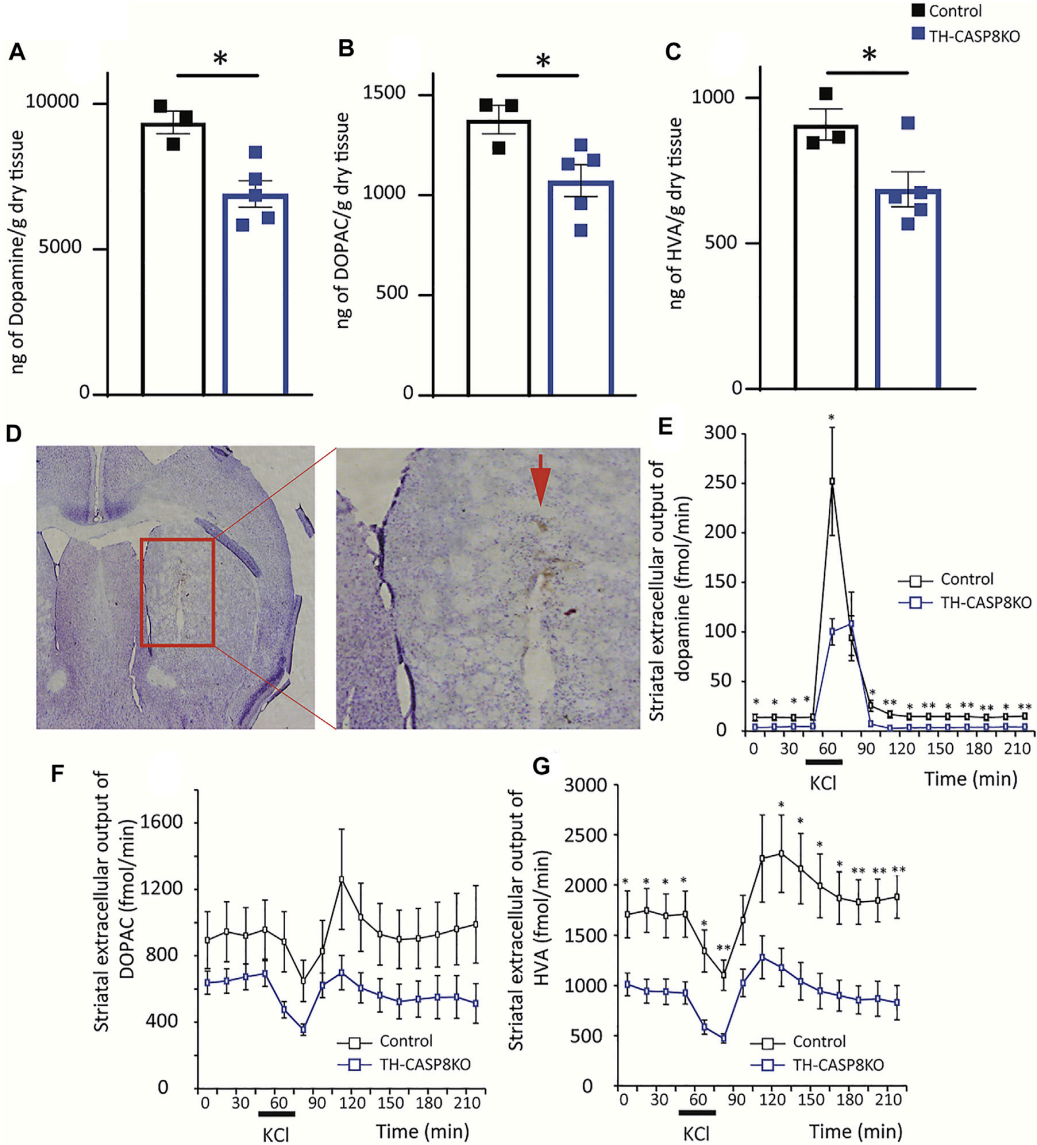
Effect of *CASP8* deletion on DA and its metabolites levels in the striatum. Quantification by HPLC of DA **(A)** and its metabolites DOPAC **(B)** and HVA **(C)** in the striatum of control and TH-CASP8KO animals. **(D)** Coronal section showing the place where the microdialysis recording cannula was located (red arrow). Scale bar 400 and 150 µm. Effect of 60 mM potassium perfusion on extracellular levels of DA **(E)**, DOPAC **(F)**, and HVA **(G)** in the striatum of control and TH-CASP8KO animals. Data are expressed as mean ± SEM of n = 6-7 mice/group. The two-tailed unpaired *t* test was used.*, *p* < 0.05; **, *p* < 0.01 *versus* control mice.

The presence of fewer TH^+^ fibers in the striatum of TH-CASP8KO mice could indicate that DA and its metabolites (DOPAC and HVA) are less available to be released. First, using the HPLC approach, DA, DOPAC, and HVA were measured in fresh striatum tissues, and we found that the neurotransmitter level declined in conditional mice compared to controls (∼22%; Figure 3A; *p* < 0.05). Furthermore, its metabolites were also reduced in similar proportions (Figures 3B,C; *p* < 0.05). Second, we checked if the release of DA was compensated or if this lower DA availability was accompanied by a decline of DA release. For this purpose, microdialysis was performed in the striatum in all experimental groups to measure extracellular levels of DA and its metabolites after potassium-induced release (Figures 3D–G). A reduction in basal striatal levels of DA, and HVA in TH-CASP8KO animals compared to controls was found (Figures 3E,G; *p* < 0.05). Moreover, the extracellular output of DA increased in both groups in response to the perfusion of KCl 60 mM (Figure 3E), being the DA level in TH-CASP8KO mice lower than in the control (Figure 3E, *p* < 0.05). Accordingly, the extracellular output of the HVA metabolite in response to the perfusion of 60 mM KCl decreased in both groups (Figure 3G). However, no differences in DOPAC release were found between both groups (Figure 3F). This analysis demonstrates striatal dopaminergic hypofunction in terms of DA release and basal extracellular levels. Together, all these data suggest that the conditional depletion of *CASP8* in TH neurons affects the dopaminergic system, which is incapable to maintain normal DA levels in the striatum.

### 3.4 The Deletion of *CASP8* in TH Neurons Alters the Number of Synapses in the Caudate Nucleus of TH-CASP8KO Mice

**FIGURE 4:**
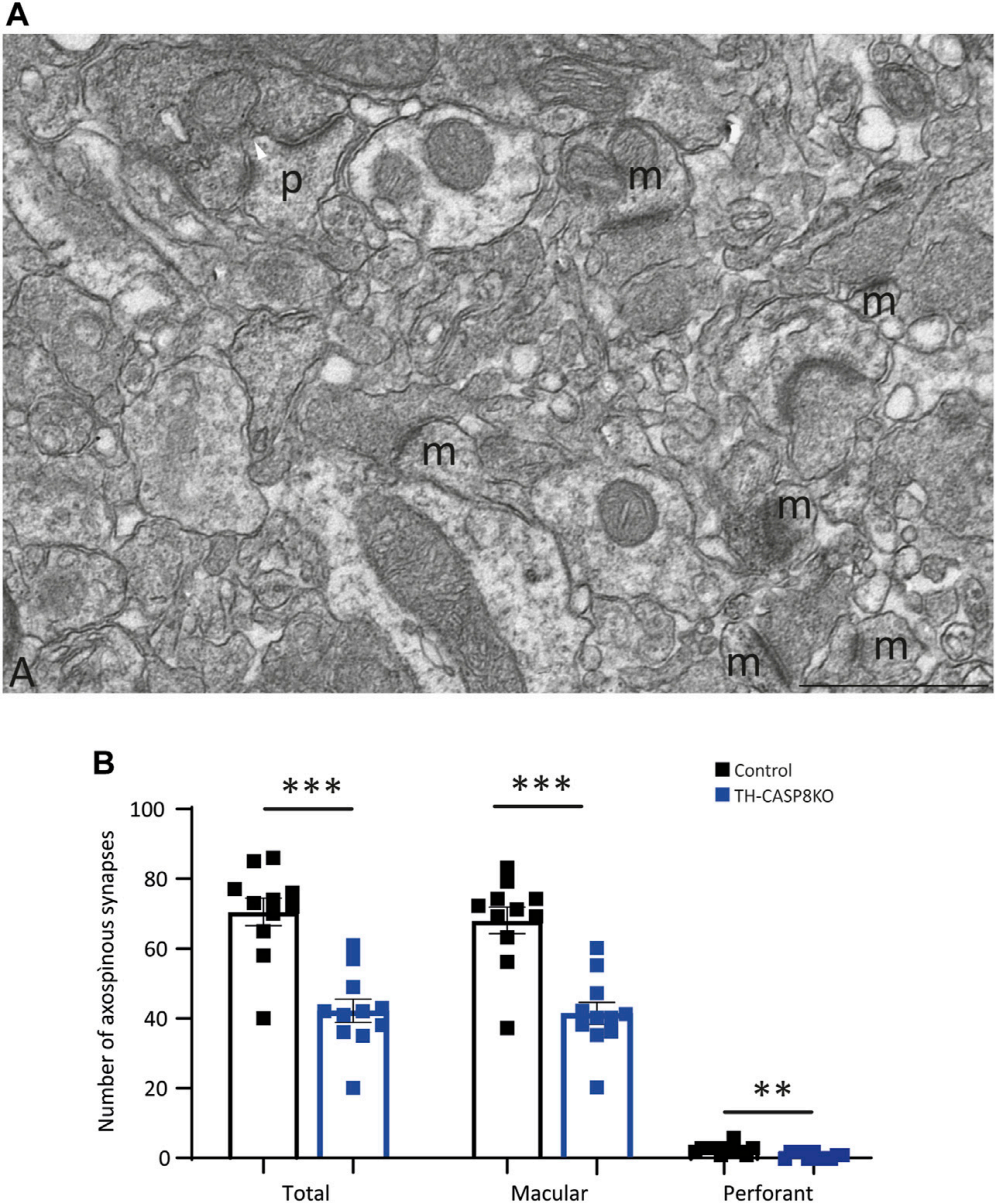
Synapse types in the striatum. **(A)** Microphotograph of a region of 17.7 µm^2^ taken from one of the striatum mosaics used for counting axospinous synapses. Two types of axospinous synapses were found: (i) the most abundant were the macular synapses characterized by a dense, continue and straight postsynaptic membrane (m), and (ii) the perforated synapses in which the central zone of the postsynaptic membrane loses its thickening and invaginates within the presynaptic terminal (p). Scale bar = 1 µm. **(B)** Quantitative analysis shows that the number of striatal axospinous synapses was higher in the control than in the TH-CASP8KO mice. T, total number of synapses, M, number of macular synapses. P, number of perforated synapses, **, *p* < 0.01; ***, *p* < 0.001.

Once dopaminergic hypofunction was described in the midbrain in TH-CASP8KO, the architecture of the striatal synapses was studied by electron microscopy. Striatal neurons have been morphologically classified according to the presence or absence of spines in their dendritic trees in two main types: medium spiny neurons and aspiny neurons (Bolam, 1984). Medium spiny neurons are the basic component of the two striatal outputs of the cortex-striatum-thalamus-cortex loop: the indirect GABA pathway projecting to the globus pallidus and the direct GABA pathway projecting to the SN (Bolam et al., 2000). The axons of the corticostriatal and thalamostriatal circuits establish asymmetric synapses with the spines of medium spiny neurons (Arbuthnott et al., 2000) whose activity is modulated by dopaminergic (Gerfen and Surmeier, 2011) and cholinergic striatal inputs (Calabresi et al., 2000). As in other parts of the CNS, the spines of the striatal neurons are the morphofunctional relationship of synaptic plasticity, varying their number and morphology according to changes in their inputs in both normal and pathological conditions (Du and Graves, 2019), thus being associated with the striatal learning (Iino et al., 2020). Therefore, we counted the number of striatal axospinous synapses as an index of the putative effect that changes in DA release in TH-CASP8KO mice throughout the life of animal life could exert on the striatal circuits. According to the morphology of the postsynaptic partner, two types of axospinous synapses can be found. The macular synapses in which the active zone faces a straight postsynaptic density (Figures 4A,M) and the perforated synapses in which the central part of the postsynaptic membrane is devoid of thickening and invaginates into the presynaptic terminal (Figures 4A,P) (Petralia et al., 2018); see also (García-Domínguez et al., 2021). To avoid biased counts, the size of the striatal neuropil areas selected for counting was similar (control 3,057.81 μm^2^ mean = 277.98 ± 22.57 μm^2^ *versus* TH-CASP8KO 3043.26 μm^2^ mean = 276.66 ± 37.64 μm^2^; *p* = 0.92). Quantification of axospinous synapses showed a decrease in the number of axospinous synapses in the TH-CASP8KO striatum relative to the control. This difference was statistically significant in the total number of synapses (Figure 4B, Total; *p* < 0.001), as well as in the number of macular synapses (Figures 4B,N; *p* < 0.001) and perforated synapses (Figure 4B, P; *p* < 0.01). 25% of the striatal dopaminergic inputs form axospinous synapses (Descarries et al., 1996). A naïve explanation would be that the decrease from 75% to the 40% of the total of axospinous synapses observed here (Figure 4B) is due to the absence of dopaminergic striatal innervation; however, since this loss is not complete (Figures 2E,F), this explanation can easily be excluded. It has been clearly established that dopaminergic presynaptic terminals ending in the neck spine’s are convergent to glutamatergic striatal inputs (for a review see Morales and Pickel, 2012). Furthermore, dopamine released at these axospinous synapses could act through spine D1-D2 receptors (Morales and Pickel, 2012, page 78). Therefore, the decrease in axospinous synapses could be the reflect of the effects of the development of striatal glutamatergic circuits under low DA levels along the life from birth. In fact, several behavioral alterations and loss of synaptic spines have been reported in the DA transporter (DAT)KO mice (Berlanga, et al., 2011).

### 3.5 TH-CASP8KO Mice Exhibit Novelty-Induced Hyperlocomotion, Repetitive and Ritualistic Behaviors

**FIGURE 5:**
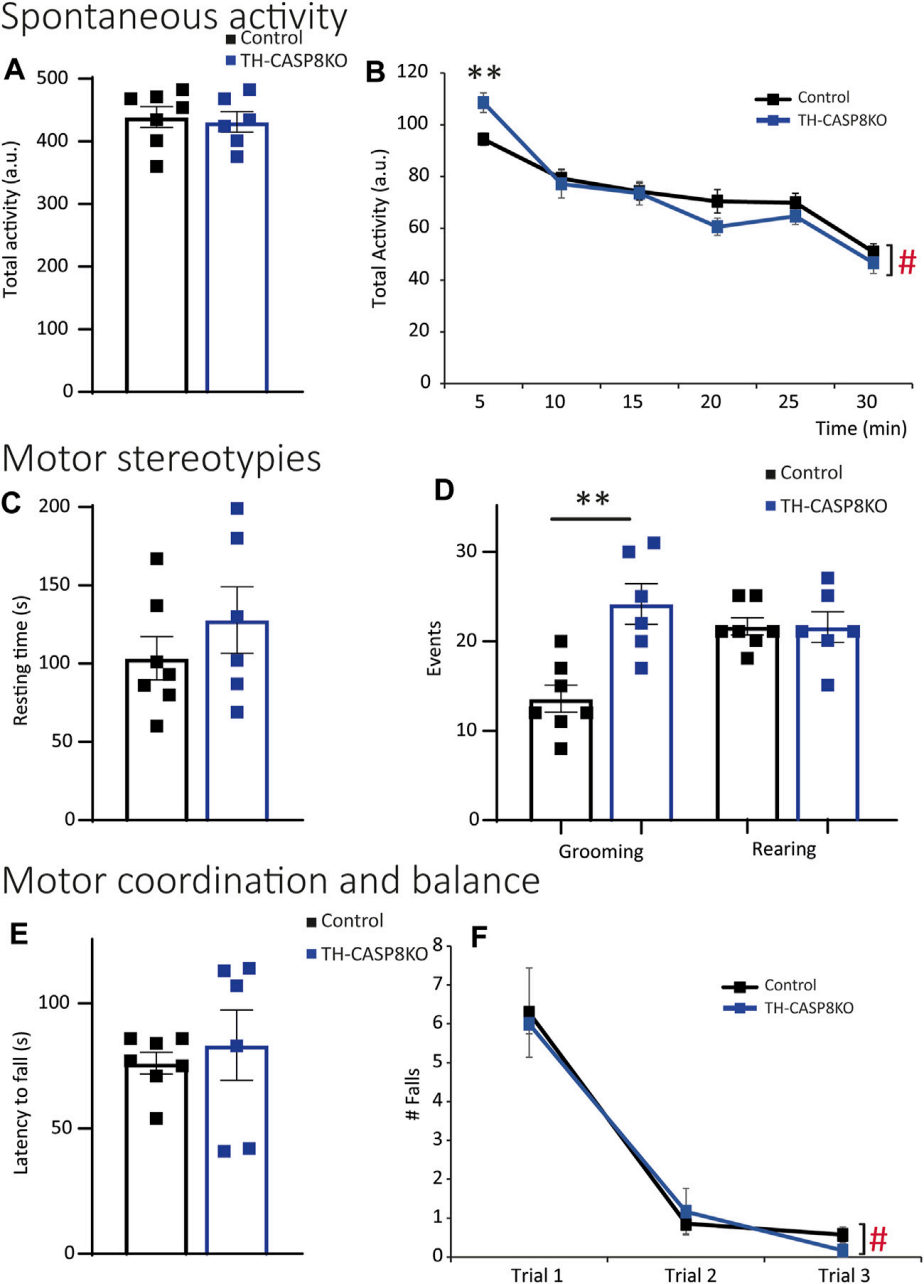
TH-CASP8KO mice are hyperlocomotive in the face of novel context and exhibit repetitive and perseverant behaviors. **(A,B)** TH-CASP8KO mice show similar activity for 30 min (total activity; **(A)**) and for each time bin except for the first 5 min (every 5 min; **(B)**) in the open field test. TH-CASP8KO mice show the same resting time during the open field session **(C)**, however, spending more time grooming but not rearing than control animals **(D)**. Their rotarod performance is comparable to the control group, in both the accelerated rotarod (4–40 rpm; **(E)**) and the constant velocity rotarod (5 rpm; **(F)**). Data are expressed as mean ± SEM of n = 6–7 mice/group. The two-tailed unpaired *t* test was used **(A,C–E)**. ** (Control *vs*. TH-CASP8KO), *p* < 0.01; # (Control and TH-CASP8KO 5 min vs. Control and TH-CASP8KO 30 min), *p* < 0.001 *versus* control mice. One-way analysis of variance (ANOVA) followed by Bonferroni post hoc for multiple comparisons **(B,F)** was used. a. u.: arbitrary units.

**FIGURE 6:**
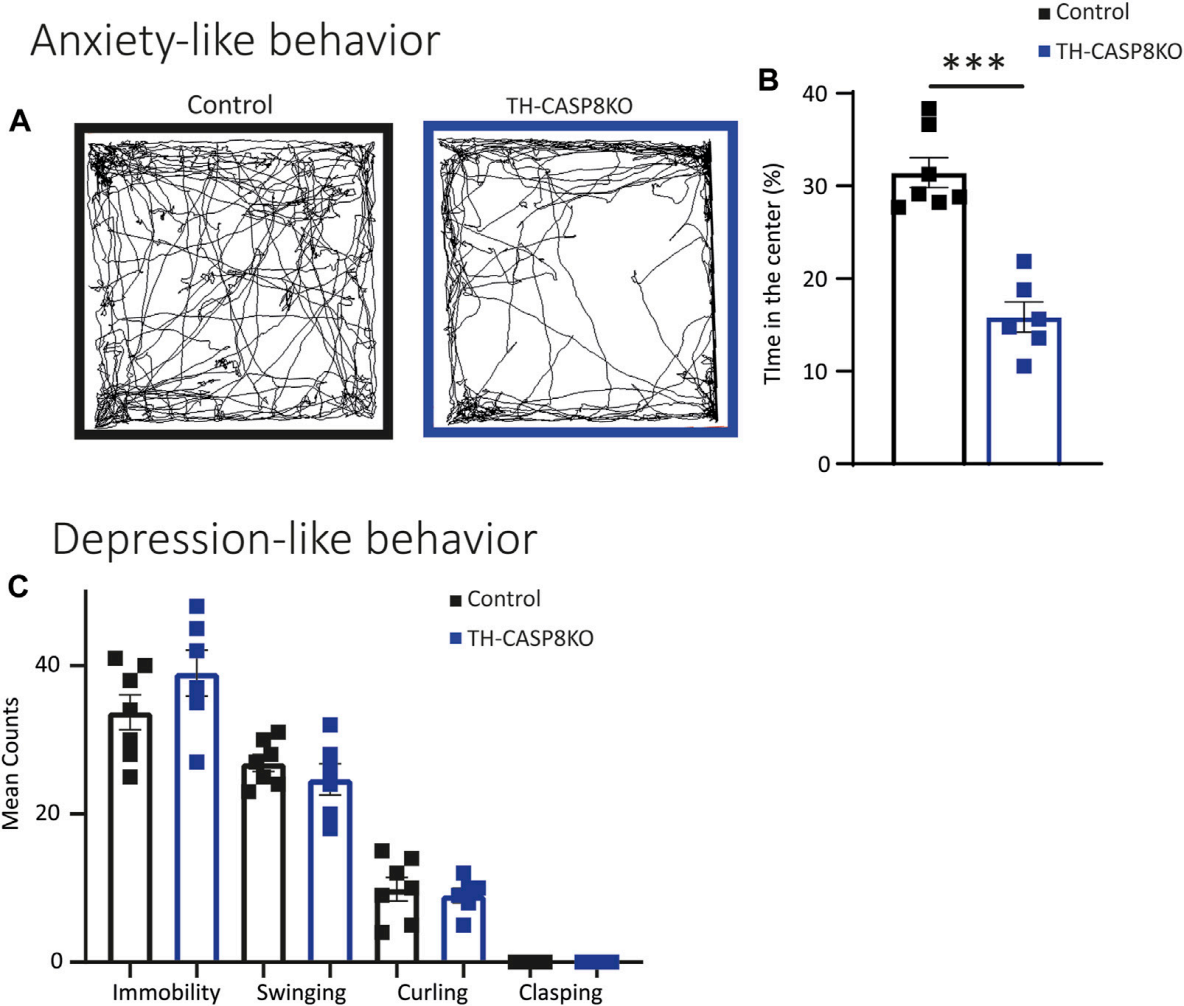
TH-CASP8KO mice exhibit anxiety-like behavior but not depressive-like behavior. **(A–B)** TH-CASP8KO spend more time in the open field periphery than in the center. **(A)** Representative traces of the open field activity and **(B)** quantification, expressed as a percentage of time spent in the center **(B)**. TH-CASP8KO mice show immobility, clasping, swinging, and curling similar to control mice in tail suspension test **(C)**. Data are expressed as mean ± SEM of n = 6–7 mice/group. The two-tailed unpaired *t* test was used.***p < 0.001 *versus* control mice.

Decrease in TH^+^ synapses and in the DA release in striatum of TH-CASP8KO mice could lead to motor behavior impairments. To test this, a wide range of behavior paradigms was performed. Although spontaneous total locomotor activity was similar in TH-CASP8KO mice compared to control animals (Figure 5A), during the first 5 mins the activity in conditional mice was augmented ∼15% (Figure 5B; *p* < 0.01). Interestingly, motor activity did not increase with time compared to control mice (Figure 5B). Therefore, TH-CASP8KO mice showed correct habituation to the context. However, the resting time was similar between the groups throughout the duration of the test (30 min) (Figure 5C). Second, in this test, the characteristic stereotype in mice was measured, finding an increase in grooming but not in rearing movements in TH-CASP8KO mice *versus* the control group (Figure 5D; *p* < 0.01 and *p* > 0.05 respectively). To test whether motor alterations induced impairment of motor coordination and balance, the rotarod was performed. The results reported that motor coordination and balance were similar in TH-CASP8KO and control mice for accelerated (4–40 rpm; Figure 5E) and constant velocity (5 rpm; Figure 5F) in the rotarod test. Therefore, the increased in the activity at the start of the locomotor test together with an increased in stereotypes in TH-CASP8KO may also be linked to anxiety- and/or depression-like conduct. To investigate this behavior, the time spent in the center of the open field arena compared to the margin was measured, being ∼50% lower in TH-CASP8KO than in control mice (Figures 6A,B; *p* < 0.001). Additionally, after performing the tail suspension test (Figure 6C), TH-CASP8KO mice showed similar immobility, swinging, curling, and clasping score than control mice (Figure 6C). Altogether, these data indicated that TH-CASP8KO mice exhibited novelty-induced hyperlocomotor activity and altered behavior associated with typical anxiety-like behavior.

### 3.6 TH-CASP8KO Mice Show Typical Responses to Sensory Stimuli

**FIGURE 7:**
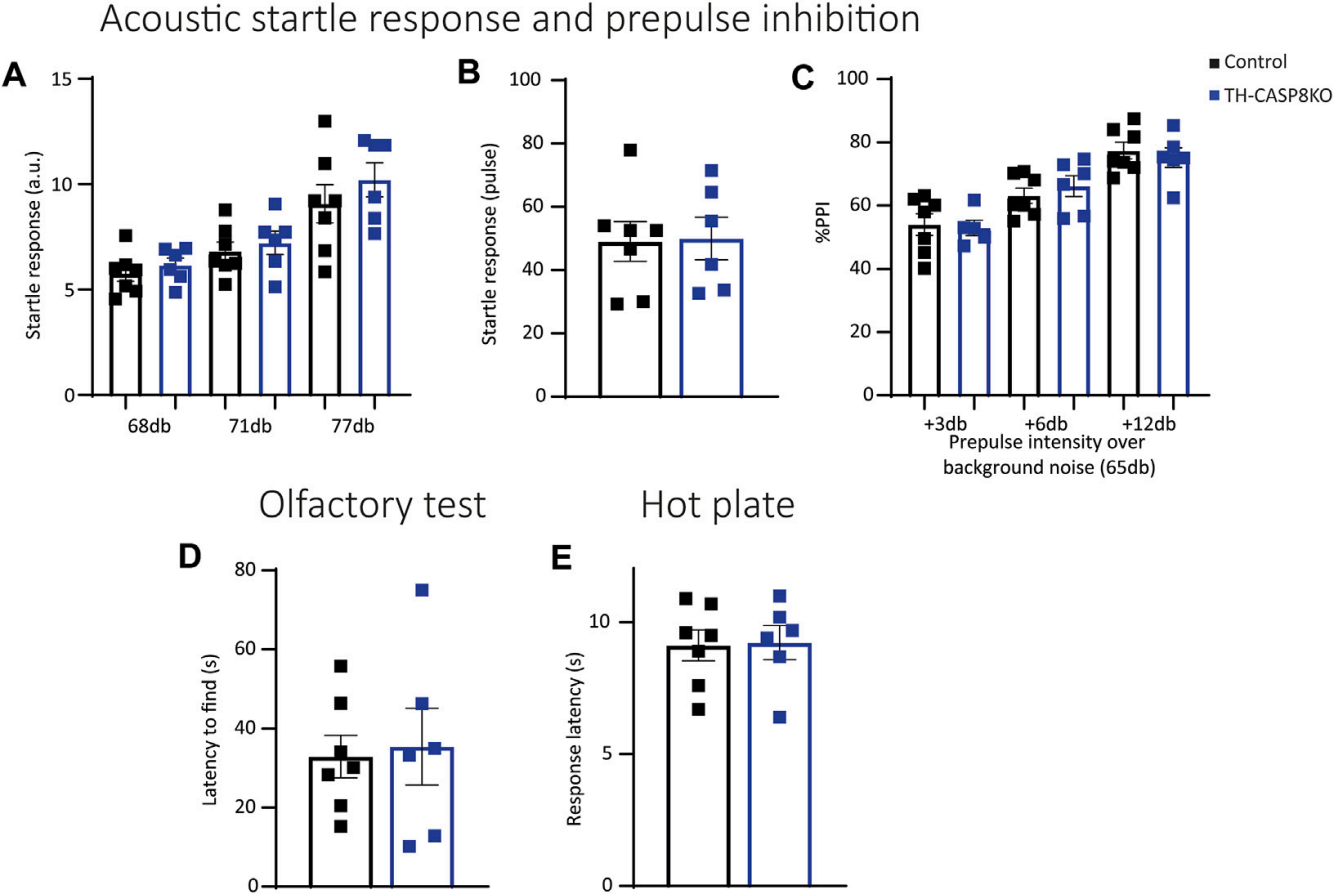
TH-CASP8KO mice exhibit normal responses to sensory stimuli. **(A)** No significant differences were detected at all decibel levels in acoustic startle responses to low-intensity pulses in TH-CASP8KO mice. They showed no differences in the auditory startle response to a 120 dB broadband noise pulse **(B)** or in prepulse inhibition (PPI; when prepulses were delivered at intensities of 3, 6 and 12 dB above a background noise of 65 dB) compared to control mice **(C)**. **(D)** TH-CASP8KO mice displayed a similar latency to uncover buried food in olfactory tests with respect to control mice. **(E)** The response on hot plate in TH-CASP8KO mice and controls measured as mean latency time to jump, lick, or vocalize was similar. Data are expressed as mean ± SEM of n = 6–7 mice/group. The two-tailed unpaired *t* test was used.

Repetitive conduct could be associated to sensorimotor gating impairment. To check this, the acoustic startle response (ASR) and PPI ratio were measured in experimental groups. PPI measures the attenuation of a reflexive startle response when starting stimulus is delivered after a weak prestimulus (prepulse). No significant differences at different decibel levels were detected at low intensities pulses (prepulse intensities) in TH-CASP8KO mice (Figure 7A). Furthermore, no differences in auditory startle response to a 120 dB broadband noise pulse (Figure 7B) or in PPI (when prepulses were delivered at intensities of 3 dB, 6 dB, and 12 dB above a background noise of 65 dB) were found compared to control mice (Figure 7C). In the same way, TH-CASP8KO mice displayed similar latency to discover buried food in the olfactory test (Figure 7D) and a similar thermal threshold, indicated by equal in time response latency in a hot plate (Figure 7E). These results may indicate that the reaction to the aversive sensory experience in TH-CAS8KO mice was similar to that in the control group.

### 3.7 TH-CASP8KO Mice do Not Exhibit Altered Social Interaction and Nesting Disability

**FIGURE 8:**
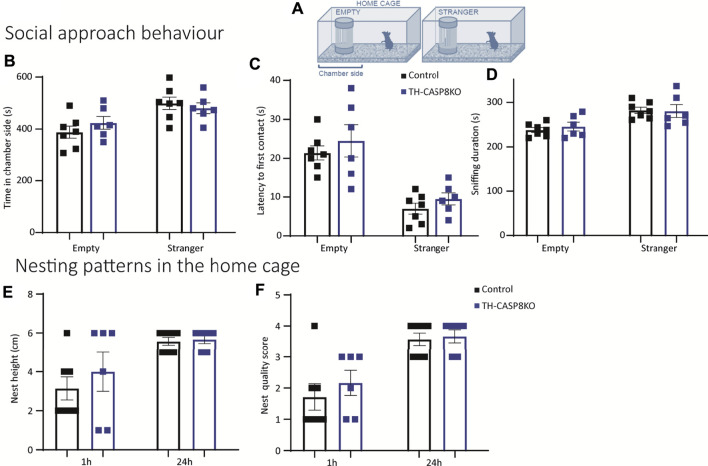
Social interaction and nesting ability in TH-CASP8KO mice. **(A)** Diagram of a test box (home cage) containing a small compartment that is empty or occupied by stranger mice (from a different strain). **(B)** TH-CASP8KO mice with a similar time in close proximity to the object (empty compartment) or the stranger mouse (time in chamber side), and an equal time away from it than the control group. Similarly, no differences were found in the latency to first contact **(C)** and in the duration of sniffing the object or the stranger mouse between the experimental groups **(D)**. The height **(E)** and quality score **(F)** of the nest was similar in TH-CASP8KO and control mice at 1 h and at 24 h. Data are expressed as mean ± SEM of n = 6–7 mice/group. The two-tailed unpaired *t* test was used.

*Mus musculus* is a highly social species. Since the previously described behavioral alterations could lead to a change in social behavior in the conditional model, it was necessary to explore the social interaction in the experimental mice. First, a modified home cage was used to test the social approach behavior; the home cage contained a wire cage, empty or occupied by stranger mouse of a different strain (Figure 8A). TH-CASP8KO mice spent analogous time than the control group both in close proximity to the empty wire cage and when the wire cage contained a stranger mouse (Figure 8B). Similarly, the latency to first contact and the time spent sniffing were similar in TH-CASP8KO mice than in the control group either when the compartment was empty or when it was occupied with a stranger mouse (Figures 8C,D). Second, nesting patterns in the home cage were studied, as changes could indicate impairment in social behavior. Height (Figure 8E) and quality score (Figure 8F) of the nesting were comparable in TH-CASP8KO at 1 and 24 h. Taken together, these results indicate that the absence of CASP8 in TH neurons did not alter social behavior.

## 4 Discussion

Since ASD is a highly prevalent disorder without effective treatment, there is an urgent need to elucidate the molecular basis that governs this complex disease. Here, we show that the specific deletion of *CASP8* in the dopaminergic system leads to alterations in the nigrostriatal pathway, including an increase in the number of TH positive neurons in SNpc. Neurochemical and anatomical analysis of TH-CASP8KO mice demonstrated general dopaminergic hypofunction that finally led to behavioral patterns partially compatible with ASD. These results revitalize the importance of caspases in the development and wiring of the dopaminergic system and shed light on the implication of this system in the etiology of ASD.

The development of dopaminergic neurons in the midbrain starts around day 8 of embryonic development in the mouse with induction of the ventral midbrain, followed by the specification of a distinct dopaminergic progenitor domain that allows differentiation, proliferation, and neurogenesis (Blaess and Ang, 2015). During differentiation, dopaminergic neurons migrate away from the ventricular zone into the mantle layer, finally acquiring a mature dopaminergic phenotype and forming the three distinct groups of dopaminergic neurons, SNpc, VTA, and the retrorubral field, and establish axonal projections and synapses (Blaess and Ang, 2015). Physiological clearance of unnecessary neuronal axons and synapses is carried out through the apoptotic pathway/machinery (Jin et al., 2019).

The involvement of caspases in brain shaping has been consistently demonstrated (Yuan and Yankner, 2000). As cell death mediators, they clearly define the size of the cell population of developing embryos. Recently, caspases have also been shown to promote not only induction of apoptosis, but also the development of neurons, including branching of the axon and synapses formation and maturation of synapses (Nguyen et al., 2021).

We have previously demonstrated that selective deletion of *CASP3* in TH-expressing cells confers dopaminergic hyperinnervation in the nigrostriatal and mesocorticolimbic circuits and striatal DA hypofunction with abnormal motor behavior. Interestingly, TH-CASP3KO mice exhibited prominent autistic-like behavior, including motor and social alterations, thus rising as a promising animal model for ASD (García-Domínguez et al., 2021).

As stated above, CASP3 can be activated by the intrinsic pathway or by the extrinsic pathway, with CASP8 being the best known initiator caspase involved in this latter mechanism. In this work, we have analyzed the possible involvement of CASP8-associated pathways in the development of the dopaminergic system of the midbrain and its involvement in the etiology of ADS. Therefore, we took advantage of conditional TH-CASP8KO mice specific for catecholaminergic neurons. Considering that the organization of the dopaminergic system in humans and mice is similar, we provide a new animal model that may be relevant to study the development of dopaminergic neurons. (Blaess & Ang, 2015). ASD has been shown to affect 2 to 3 times more men than women. Therefore, studying sex differences in ASD is an interesting issue. In fact, there are more than 80 papers in PubMed on sex influence in different rodent models of ASD (for example, Gouda et al., 2022; Ferreira et al., 2022; Arnold and Saijo, 2021). For this reason, we have performed all our experiments in male, since mixing both sexes surely would arise a high deviation in the data. Using the TH-CASP8KO model, we found an increase in the number of TH^+^ neurons and increased TH mRNA levels in SNpc in adult animals to a degree similar to that seen in TH-CASP3KO animals, suggesting that this effect may be mediated by CASP8. These changes were accompanied by a reduction in TH density in the striatum and a subsequent reduction in potassium-evoked DA release and basal extracellular DA levels in TH-CASP8KO mice, clearly showing low dopamine functioning in the nigrostriatal system. Furthermore, the decrease in axospinous synapses will be the functional substrate of this hypofunctional striatum in TH-CASP8KO. These data are in line with those found in animals lacking *CASP3*, thus highlighting the possibility that CASP8 activation could act upstream to trigger caspase-3 cleavage. This analysis is consistent with the apoptotic death of nigral dopaminergic neurons in early postnatal development, which is regionally specific (Jackson-Lewis et al., 2000). In fact, during this period, the selective death of nigral dopaminergic neurons models the midbrain to clearly define SNpc (Jackson-Lewis et al., 2000), which was not fully present in TH-CASP8KO mice. However, this cannot be the only pathway related to CASP3 activation, since in TH-CASP8KO animals and contrary to what seen in TH-CASP3KO mice, we could not find any difference in VTA, a structure related to the mesocorticolimbic system.

To study how abnormalities in the nigrostriatal system in animals lacking CASP8 affect behavior, we performed a series of behavioral tests to analyze relevant functions attributed to DA, including motor activity, motivation, reward processing, and social interaction (Wise, 2004). This analysis revealed that our experimental mice exhibited motor stereotypies, one of the core symptoms of ASD. Stereotypical behaviors observed in autistic patients have been described to arise from a dysfunction of the nigrostriatal pathway, which has been shown to be involved in mediating entrapment into these loops of purposeless stereotyped patterns of behavior (Lewis and Kim, 2009), (Pavǎ;l, 2017). Animals lacking CASP8 also exhibit anxiety behavior, which is one of the most common co-occurring psychiatric conditions in children with ASD (Simonoff et al., 2008). However, TH-CASP8KO animals do not show impairments of social interaction, which could be related to the absence of abnormalities in VTA, a core region of the mesocortical pathway. This is a remarkable finding, as it perfectly fits the hypothesis that suggests a determining role for mesolimbic DA signaling anomalies in the generation of the social characteristics of ASD (Simonoff et al., 2008).

Collectively, these results highlight the implication of CASP8 signaling in the early development of the dopaminergic system. As stated above, this pathway can be triggered by binding of different ligands, such as tumor necrosis factor (TNF), FasL/CD95L, and Apo2L/TRAIL, to its receptor on the target cell surface (Cassatella et al., 2006; Ehrlich et al., 2003; Halaas et al., 2000). This raises the question of the probable expression of one or more death ligands during embryonic dopaminergic development. *CASP8* gene deletion leads to embryonic lethality accompanied by failure of yolk sac and hematopoiesis, an observation that supports the role of CASP8 in cellular processes beyond apoptosis during embryonic development (Varfolomeev et al., 1998). Subsequent work demonstrated that concurrent ablation of CASP8 and RIPK3 prevented development defects associated with CASP8 deficiency (Oberst et al., 2011; Kaiser et al., 2011). Since RIPK3 drives necroptosis, these studies highlighted an important survival role for CASP8 as a suppressor of the necroptotic process (Weinlich et al., 2017). Since we performed a selective deletion of the *CASP8* gene within catecholaminergic neurons, the dopaminergic hypofunction seen under our experimental conditions could be explained by necroptosis. In fact, necroptosis has been reported to occur in dopaminergic neurons of the midbrain in response to MPTP administration, an animal model of Parkinson´s disease (Hu et al., 2019; Iannielli et al., 2018). However, the aberrant number of dopaminergic neurons in CASP8KO mice argues against this possibility. Different scenarios may alternatively explain the phenotypic characteristics observed in the dopaminergic system. Fas mRNA and protein are transiently expressed in close proximity to FasL-expressing cells within the developing subventricular zone, the starting point of the development of dopaminergic neurons, during the peak period of apoptosis (Cheema et al., 1999). Besides, TNF-α mRNA is expressed in the developing brain of mice (Burns et al., 1993; Deverman and Patterson, 2009), sheep (Dziegielewska et al., 2000) and goose (Song et al., 2012), thus supporting the role of TNF-α. In fact, TNFR1 is expressed in most cell types (McCoy and Tansey, 2008) and can be internalized after TNF-α binding, which finally leads to the recruitment of pro-CASP8 (Micheau and Tschopp, 2003); (Schneider-Brachert et al., 2004). Since TNF-α expression is restricted mainly to glial cells (McCoy and Tansey, 2008), new studies are required to evaluate the roles of these cells in the regulation of wiring and survival of the dopaminergic system and the potential contribution of TNF-α and CASP8. Indeed, 1) mice deficient in Pu.1, which disrupts microglia generation, allowed identification of a role for microglia in the growth of dopaminergic axons in the forebrain (Squarzoni et al., 2014), and 2) recent RNAseq analysis of microglia at the single cell level has revealed that the highest microglial heterogeneity is observed during the late embryonic and early postnatal periods (Stratoulias et al., 2019).

Taken together, our study supports the participation of CASP8 in the regulation of the fate of nigral dopaminergic neurons. Our study also revitalizes the implication of the dopaminergic system in the etiology of ASD and delve into the biology of this disorder, providing a mild model ASD. These observations are consistent with current therapy for ASD, as the few treatments that aim to alleviate symptoms of this disease are precisely targeting the dopaminergic system. These treatments include the partial D2 agonist aripiprazole (Bartram et al., 2019) and D2 antagonist risperidone (McCracken et al., 2002). Our results open up an exciting new therapeutic field, as, interestingly, alterations in spine density are observed in several regions of patients with ASD, focusing future therapeutic strategies on the synaptic spine itself (for a review, see Bagni and Zukin, 2019; Varghese et al., 2017). Therefore, the decrease in excitatory synapses found here was correlated with a decrease in the density of striatal axons, possibly due to developmental axonal pruning, as those described by Tang et al. (2014). In conclusion, the identification of chemoattractants and axon pruning mechanisms in the determination of nigrostriatal and mesocortical pathways would help to understand the final development of dopaminergic connectivity of the midbrain and will likely lead to the discovery of new promising avenues of research on the etiology of various neurological and neurodevelopmental disorders, including ASD.

## Data Availability

The raw data supporting the conclusion of this article will be made available by the authors, without undue reservation.
