# Erratum: The absence of caspase-8 in the dopaminergic system leads to mild autism-like behavior

**DOI:** 10.3389/fcell.2022.1040623

**Published:** 2022-09-28

**Authors:** Frontiers Production Office

**Affiliations:** Frontiers Media SA, Lausanne, Switzerland

**Keywords:** autism spectrum disorder, dopaminergic system, caspase 8, electron microscopy, synapsis, behavioral test

Due to a production error, the authors’ surnames and first names were incorrectly switched in the citation, which should read as follows:

Suárez-Pereira I, García-Domínguez I, Bravo L, Santiago M, García-Revilla J, Espinosa-Oliva AM, Alonso-Bellido IM, López-Martín C, Pérez-Villegas EM, Armengol JA, Berrocoso E, Venero JL, de Pablos RM and Ruiz R.

The publisher apologizes for this mistake.

The original version of this article has been updated.

